# Severe COVID-19 with acute respiratory distress syndrome (ARDS) in a sickle cell disease adult patient: case report

**DOI:** 10.1186/s12890-021-01412-x

**Published:** 2021-01-29

**Authors:** Marion Teulier, Alexandre Elabbadi, Grigorios Gerotziafas, François Lionnet, Guillaume Voiriot, Muriel Fartoukh

**Affiliations:** 1 Sorbonne Université, Assistance Publique – Hôpitaux de Paris, Service de médecine intensive réanimation, Hôpital Tenon, Paris, France; 2 Sorbonne Université, Assistance Publique – Hôpitaux de Paris, Unité d’Explorations Fonctionnelles et Génétiques du Risque Vasculaire, Consultation Thrombose – Oncologie, Hôpital Tenon, Paris, France; 3 Sorbonne Université, Assistance Publique – Hôpitaux de Paris, Service de médecine interne, Hôpital Tenon, Paris, France

**Keywords:** Sickle cell disease, COVID-19, ARDS, ECMO, Case report

## Abstract

**Background:**

Sickle-cell anaemia is a widespread genetic disease prevalent worldwide among African and African-American populations. The pathogenesis is most often revealed by pulmonary conditions, including acute thoracic syndrome, which is affecting the life expectancy of these populations. The global spread of CoV2-SARS infection with a respiratory tropism, endothelial damages and procoagulant status endangers the SCD population. However, with only a few case reports, consequences of the Covid-19 pandemic on SCD population remain poorly known.

**Case presentation:**

We report a case of a 33-year-old man with a history of homozygous SS homozygous sickle cell anemia who consulted on March 24, 2020 for febrile dyspnea 11 days after the onset of symptoms. A nasopharyngeal swab was positive for SARS-CoV-2. His respiratory status worsened rapidly in the emergency room and then in ICU leading to severe ARDS requiring intubation, curarization, and venovenous ECMO. Hematologically, severe hemolysis associated with major thrombocytopenia without documented spinal cord injury was noted. Several transfusion exchanges are performed. The evolution was finally slowly favorable and led to discharge from the intensive care unit and then from the hospital.

**Conclusions:**

This case recalls the importance of an increased prevention policy against COVID-19among the SCD population. In addition, from a therapeutic point of view, it advocates (1) a high preventive anticoagulation from the outset according to the level of D-dimers (2) the use of venovenous ECMO in this particular case, whereas this technique has had rather disappointing results in acute chest syndromes. (3) Unexpectedly, our patient did not develop pulmonary arterial hypertension (PAH) and acute cor pulmonale (ACP), whereas this is a common feature of ARDS during SCD. These last two observations suggest a different pathophysiology of pulmonary disorders in SCD patients in the case of SARS COv2. It could be associated with marked hypoxemia secondary to pulmonary vascular vasodilation.

## Background

Sickle cell disease (SCD) is a serious monogenic disorder that reduces life expectancy. The acute chest syndrome (ACS) is one of the most frequent condition requiring hospitalization in SCD patients, often with an infectious event as the initial cause, and the leading cause of death among SCD adult patients [1]. Consequences of the Covid-19 pandemic on SCD population remain poorly known, with only a few case reports, including one non-severe case in a 21‐year‐old man hospitalized with a history of SCD (HbS/β0‐thalassemia) on maintenance hydroxyurea therapy [2]; a series of four clinical cases of patients from 22 to 41 years old with ACS [3]; two cases of ACS [4]. Another case describes a patient with pneumonia and severe ACS successfully treated with Tocilizumab [5]. None of these patients was reported to develop acute respiratory distress syndrome (ARDS).

## Case presentation

### Patient information

A 33-year-old man with a history of SCD and moderate overweight (BMI, 28.4 kg/m^2^) presented on March 24, 2020 to the Emergency Department of Tenon hospital (a referral SCD center in Paris, France), for a febrile dyspnea. Sickle cell homozygous disease (baseline hemoglobin [Hb] value 11.5 g/dl, HbF 2.5%, HbS 87%) was known since childhood, and he had suffered mild episodes of vaso-occlusive crisis, and two episodes of ACS. Red blood cell transfusions had been required only once in the previous 20 years. Regular follow-up of SCD at our center had revealed early ophthalmological (Goldberg's stage III retinopathy) and renal (GFR 120 mL/min and microabuminuria at 0.75 g/L) damage.

### Clinical findings

**Fig. 1:**
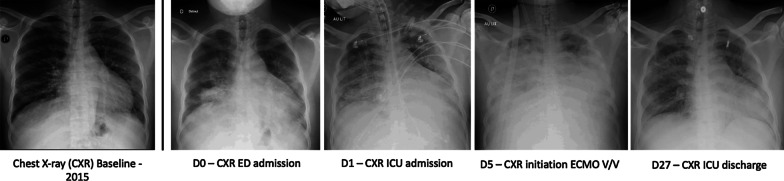
Chest X-ray (CXR) evolution through hospitalization

The symptoms first appeared 11 days before admission with cough and headache. Then, he developed fever and dyspnea. In the Emergency Room, physical examination revealed fever (39 °C) and signs of acute respiratory distress (RR 36 cycles/min, room air pulse oximetry 88%), without hemodynamic instability (BP 134/82 mmHg, HR 106/min). The patient had no history of recent medication, he reported a contact with his sick girlfriend. Chest X-ray showed bilateral alveolar and interstitial infiltrates predominating in the middle lobe (Fig. 1). Blood analysis revealed moderate anemia, reticulocytopenia, moderate hemolysis, lymphocytopenia and a major inflammatory syndrome (Table 1). Respiratory condition worsened rapidly requiring intensive care unit admission and intubation with mechanical ventilation.

**Table 1:** Biology changes through hospitalization

Day from admission		D0	D1	D5	D15	D27	D34
Variables	Normal range	ED admission	ICU admission	ECMO initiation	ECMO explantation	ICU discharge	Hospital discharge
*Blood count*							
Hemoglobin (g/dL)	12.0–18.0	11.5	10.7	10.6	8.8	10.14	7.2
Reticulocytes count (× 10.9/L)	25–125	48	38	71	361	367	331
White blood cell count (× 10.9/L)	4.0–10.0	10.5	13.86	43.29	16.37	10.14	14.79
Neutrophils (× 10.9/L)	1.7–7.0	9.44	12.29	31.64	11.63	5.57	9.73
Lymphocytes (× 10.9/L)	1.5–4.0	0.71	0.98	3.18	2.56	3,17	3.03
Platelets (× 10.9/L)	150–400	171	182	203	195	367.2	228
*Inflam*							
C reactive protein mg/L	< 5.0	300.9	343.6	303.7	160.1	63.3	20.2
Procalcitonin µg/L	< 0.5		1.78	1.56	0.38	0.59	
*Hemostasis*							
PT (%)	> 70		75	78	83	71	
Fibrinogen (g/L)	1.80–4.0		9.4	7.8	6.4	6.0	
D-dimers (ng/mL)	< 500		3030	947	> 20,000	2379	
*Hemolysis*							
ASAT (IU/L)	< 35		27	464	64	55	
ALAT (IU/L)	< 43		9	235	47	39	
Serum lactate dehydrogenase (IU/L)	125–250	393	438	2498	1044	709	
Total bilirubin (µmol/L)	< 17		24	20		25	

### Diagnosis assessment and therapeutic intervention

A nasopharyngeal swab was positive for SARS-CoV-2 at D1. Samples of respiratory tract secretions were taken on the admission day and a broad-spectrum empirical antibiotic therapy was initiated. The patient did not receive any experimental treatment specific to SARS-CoV-2.

Hemolysis worsened and prompted early blood exchange transfusions (3 sessions) for maintaining HbS value below 40%. Bone marrow aspiration was performed on D3, because of a severe ACS associated with a drop in reticulocytes and a mild thrombocytopenia suggesting a spinal thrombosis. The bone marrow aspiration demonstrated normal structure and cytology without bone marrow necrosis. The PCR search for CMV, EBV, SARS-CoV and Parvovirus-B19 were negative. On D4, as D-dimer levels had increased markedly, anticoagulation was switched from prophylactic to therapeutic dosage (intravenous unfractionated heparin with a ratio of activated partial thromboplastin time target between 2 and 2.5). As shown in Table 1, this switch was associated with significant decrease of D-Dimers. In the acute phase, a search for thromboembolic events was done through a venous doppler ultrasound of the four limbs and a transthoracic cardiac ultrasound. This exploration was initially negative. Secondarily at the time of ECMO decanulation, in a septic context, cardiac ultrasound revealed a thrombus of the inferior vena cava. Fifteen days later, i.e. one month after admission, a thoracic angioscanner was performed, which no longer revealed this thrombus.

### Follow up and outcomes

Despite neuromuscular blocking agents and repeated sessions of prone positioning, the respiratory condition progressed to severe COVID-19 with ARDS, with worsening pulmonary infiltrates (Fig. 1) and major hypoxemia (PaO_2_/FiO_2_ ratio of 54 with PEEP 10 cmH20 and FiO_2_ 100%). There were no acute cardiac nor pulmonary vascular dysfunction (explored by repeated transthoracic cardiac ultrasound). Giving this refractory ARDS situation, a venovenous extracorporeal membrane oxygenation (ECMO) was installed on D5. Because of a significant increase in inflammatory markers, and clinical and radiographic degradation, we searched for respiratory infection and we maintained concomitantly a probabilistic antibiotic treatment.

A bronchoalveolar lavage (BAL) was performed on D9 to rule out bacterial infection or fat embolism (54 mL recovered from 150 mL instilled) revealing lymphocytic alveolitis (640,000 cells with 50% macrophages, 36% lymphocytes, and 13% polymorphonuclears). The bacterial culture and the search for other viruses were negative. Blood cultures and local samples from the catheterization were negative. Antibiotic prophylaxis was suspended on D10. Secondarily to an increase in inflammatory markers and clinical deterioration, a protected distal sampling allowed the diagnosis of pneumopathy acquired under late mechanical ventilation (E. Coli and E. Cloacae), treated with the adapted antibiotics at D16.

The evolution was then slowly favorable, allowing the ECMO to be withdrawn after 10 days. The patient was extubated at D25. A laryngeal edema complicated the extubation and was treated with corticosteroid aerosol, without systemic corticotherapy and quickly resolved. Following the extubation, we did not perform non-invasive ventilation or high flow oxygen therapy. Finally, the patient was discharged from ICU on D27 and then from hospital on D34 days after his admission.

## Discussion and conclusions

The SCD population represents several million people worldwide, while there is no epidemiological data specific to this population regarding SARS-CoV-2. Although its pathophysiology and epidemiology differ from those of the H1N1 influenza epidemic, it should be recalled that in SCD patients, the severity of the infection was much greater than in the general population [6] (17% rate of hospitalization in ICU, the vast majority of whom had ARDS). The SCD population is thus likely an at-risk population regarding the Covid 19.

Distinctive features of Covid 19 appear to include endothelial damage and procoagulant status [7–9]. Indeed, Varga et al. [9] have described a shift in the vascular equilibrium with endothelitis with lymphocytic infiltration and subsequent ischemia associated with a procoagulant state, particularly in high-risk ethnicity, such as African-Americans [10]. Sickle cell patients constitutionally suffer from small vessel vasculopathy. Therefore, the SCD population seems particularly at risk of developing very severe pulmonary vascular damage during SARS-CoV-2 infection.

Experience with ECMO in SCD patients with ARDS has been disappointing so far, with a case-fatality rate of 73% [11]. The survival in our patient despite criteria for severe ARDS and refractory hypoxemia suggest that ECMO should still be considered in such patients, and that the damages associated with COVID-19 differ from ARDS due to other causes, especially ACS in SCD patients. Fogarty et al. [12] suggested that a specific phenotype of refractory ARDS caused by COVID-19 was associated with concurrent 'double-hit' pathologies targeting both ventilation and perfusion within the lungs through the involvement of ACE2 receptors present in type II pneumocytes and vascular endothelial cells.

It is noteworthy that our patient did not develop pulmonary arterial hypertension (PAH) and acute cor pulmonale (ACP), whereas this is a common feature of ARDS during SCD [13–15]. For example, Cechini et al. [13] recently reported that 100% such patients had PAH and 90% had ACP. Several echocardiographies have been performed throughout the evolution of our patient, showing no evidence of acute PAH. The lack of ACP in our patient may be related to the pathophysiology of COVID-19 and associated marked hypoxemia secondary to pulmonary vascular vasodilation [16]. Given the recorded frequency of pulmonary artery thrombosis associated with COVID-19 [17], therapeutic anticoagulation may also have contributed to a protective effect on the occurrence of ACP in our patient.

To date, there are only scarce reports of COVID-19 in SCD patients. Meanwhile, it is essential to stress the importance of increased awareness and effective preventive measures in these particular high-risk patients. The risk is indeed greater because of the increased susceptibility and severity of respiratory tropism infections but also because of the procoagulant endothelitis of the Covid-19. Therefore, compliance with preventive precautions (masks, social distancing, hand hygiene inparticular).

In terms of therapeutic intervention, early care, early blood exchanges and high preventive anticoagulation should be provided in these patients. Interestingly, ECMO should be considered and may be more effective than ACS related ARDS, due to a different pathophysiology of Covid-19 infection.

## Data Availability

All raw data (chest X-rays, biological data) are available on the APHP computer system (via a software called Orbis not remotely accessible without privileged access) and can be provided on request by the corresponding author.

## Abbreviations

SCD: Sickle cell disease
ACS: Acute chest syndrome
ARDS: Acute respiratory distress syndrome
ECMO: Extracorporeal membrane oxygenation
BAL: Bronchoalveolar lavage
ICU: Intensive care unit
PAH: Pulmonary arterial hypertension
ACP: Acute Cor Pulmonale
D: Day
ED: Emergency department
Inflam: Inflammation
